# Rheological Properties of Cemented Tailing Backfill and the Construction of a Prediction Model

**DOI:** 10.3390/ma8052076

**Published:** 2015-04-23

**Authors:** Liu Lang, KI-IL Song, Dezheng Lao, Tae-Hyuk Kwon

**Affiliations:** 1 Energy School, Xi’an University of Science and Technology, Xi’an 710054, China; E-Mail: csuliulang@163.com; 2 Key Laboratory of Western Mines and Hazards Prevention, Ministry of Education of China, Xi’an 710054, China; 3 Department of Civil Engineering, Inha University, Incheon 402-751, Korea; 4 School of Civil & Resource Engineering, University of Western Australia, Perth 6009, Australia; E-Mail: lao@civil.uwa.edu.au; 5 Department of Civil and Environmental Engineering, Korea Advanced Institute of Science and Technology (KAIST), 291 Daehak-ro, Yuseong-gu, Daejeon 305-701, Korea; E-Mail: t.kwon@kaist.ac.kr

**Keywords:** cemented tailing backfill, yield stress, viscosity, principle component analysis (PCA), back-propagation (BP) neural network

## Abstract

Workability is a key performance criterion for mining cemented tailing backfill, which should be defined in terms of rheological parameters such as yield stress and plastic viscosity. Cemented tailing backfill is basically composed of mill tailings, Portland cement, or blended cement with supplementary cement material (fly ash and blast furnace slag) and water, among others, and it is important to characterize relationships between paste components and rheological properties to optimize the workability of cemented tailing backfill. This study proposes a combined model for predicting rheological parameters of cemented tailing backfill based on a principal component analysis (PCA) and a back-propagation (BP) neural network. By analyzing experimental data on mix proportions and rheological parameters of cemented tailing backfill to determine the nonlinear relationships between rheological parameters (*i.e.*, yield stress and viscosity) and mix proportions (*i.e.*, solid concentrations, the tailing/cement ratio, the specific weight, and the slump), the study constructs a prediction model. The advantages of the combined model were as follows: First, through the PCA, original multiple variables were represented by two principal components (PCs), thereby leading to a 50% decrease in input parameters in the BP neural network model, which covered 98.634% of the original data. Second, in comparison to conventional BP neural network models, the proposed model featured a simpler network architecture, a faster training speed, and more satisfactory prediction performance. According to the test results, any error between estimated and expected output values from the combined prediction model based on the PCA and the BP neural network was within 5%, reflecting a remarkable improvement over results for BP neural network models with no PCA.

## 1. Introduction

Due to its exceptional performance, high-density unclassified tailing paste represents the future developmental direction of cemented tailing backfill as well as an inexorable trend in the promotion of “green mines” [1,2,3,4]. In this regard, an appropriate evaluation of the consistency, flowability, and workability of cemented tailing backfill is crucial for determining the ease and homogeneity with which it can be mixed, transported, placed, and compacted while avoiding the clogging or failure of pipelines transporting cemented tailing backfill. Many studies have examined the consistency, flowability, and workability of cemented tailing backfill with respect to its rheological behavior [5,6,7,8,9,10,11]. It is generally accepted that the rheological behavior of cemented tailing backfill can be approximated using the Bingham model [12], which requires two independent properties to describe the rheological behavior, including yield stress, which corresponds to the shear stress required to initiate the flow of cemented tailing backfill, and plastic viscosity, which describes paste resistance to the flow of cemented tailing backfill under some external stress [13,14].

The selection of rheological parameters is of vital importance because they are influenced by solid concentrations, the specific weight, the tailing/cement ratio, the slump, and physical/chemical properties of mill tailings (e.g., their type, chemistry, particle size distribution, and mineralogy). Traditionally, the rheological behavior of cemented tailing backfill is evaluated by the slump only from an empirical perspective without considering the theoretical level, and therefore the relationship between the slump and the rheological behavior is not reflected accurately [15,16,17,18]. As indicated in Hu [19], Christensen [20], Clayton [21], Pashias [22], and Bentz [23], models relating the slump height to yield stress have been developed for cone and cylinder slump tests, and with respect to test results from the viscometer, there is typically good agreement between predicted and test results.

Rheological properties and their prediction using the neural network have been investigated in the last two decades [24,25,26,27,28], and some scholars have used the BP neural network to predict other properties of cemented tailing backfill [29,30,31] and found simulation results for prediction models to be in good agreement with experimental results. However, the neural network has several limitations in predicting rheological properties: First, there are too many input variables (i.e., solid concentration, specific weight, tailings/cement ratio, and slump) required for predicting rheological properties, which affects the training speed of the constructed neural network. Second, input variables are highly correlated with one another, hindering the construction of a high-quality prediction model and reducing its accuracy. If it is possible to reduce the number of input variables and eliminate their correlations, then a sharp decrease in the computation time and a substantial increase in accuracy can be achieved.

Based on the aforementioned limitations of using the neural network model to predict rheological parameters of cemented tailing backfill, this study proposes a prediction model combining the principle component analysis (PCA) and the back-propagation (BP) neural network. To eliminate correlations between factors and reduce the number of input factors, the sample data were compressed by a PCA in advance not only to improve predictive accuracy but also to enhance the computational efficiency of the BP neural network while incurring almost no major change in the data. According to the results, the combined model predicted rheological parameters more precisely than the BP neural network model. The relative error was within 5%, and the predicted outcome was in good agreement with experimental results.

## 2. Cemented Tailing Backfill Properties and Their Measurement

### 2.1. Material Characterization

#### 2.1.1. Tested Tailings

Tested tailing materials were obtained from an iron mine located in the northeastern region of China. Mill tailings are main ingredients of cemented tailing backfill, and physical properties (e.g., bulk density, specific gravity, porosity, the specific surface area, and the particle size distribution) and the chemical composition play key roles in the performance of cemented tailing backfill.

Based on the *Specification of Soil Test* (SL 237-1999) [32], the bulk density and special gravity of tailings were measured using the picnometer method (SL 237-005-1999) [32] and the relative density method (SL 237-004-1999) [32], respectively. Physical properties of tailing materials are shown in Table 1.

**Table 1:** Physical properties and particle size distribution of tailings.

Physical properties		Particle size distribution	
Specific gravity	2.69	d_10_ (μm)	25.07
Bulk density (t/m^3^)	1.58	d_50_ (μm)	122.08
Porosity (%)	44.12	d_90_ (μm)	288.59
Specific surface area (m^2^/m^3^)	872,000		

**Figure 1:**
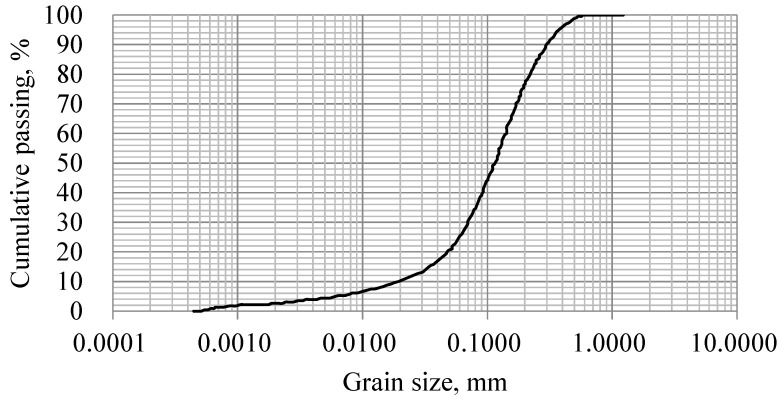
The particle size distribution of Xin-Cheng mill tailings.

As shown in Figure 1, the particle size distribution (PSD) was well graded, and *d*_10_, *d*_50_, and *d*_90_ of tailings were 25.07, 122.08, and 288.59 μm, respectively. The coefficient of uniformity of the particle size composition was 5.506. It should be noted that tailings satisfying backfilling requirements general refer to a coefficient of uniformity between 4 and 6 [33].

The main chemical elements of mill tailings are shown in Table 2. The proportions of metallic elements and related oxides (Fe, Al_2_O_3_, CaO, and MgO) were relatively high in unclassified tailings (8.85%, 4.59%, 6.78%, and 5.08%, respectively), and other metallic elements showed lower content in tailing samples. Nonmetallic elements and related oxides in mill tailings were mainly SiO_2_, S, and P (71.96%, 0.11%, and 0.07%, respectively), indicating that the low content of sulphide and phosphide had little damaging effect on the quality (e.g., strength deterioration) of cement-based backfill materials.

**Table 2:** Main chemical elements of mill tailings.

Element	Contents (%)	Element	Contents (%)
Cu	<0.005	Sn	0.058
K	1.33	Na	0.4
Pb	0.014	SiO_2_	71.96
Zn	0.037	Al_2_O_3_	4.59
Fe	8.85	CaO	6.78
Mn	0.05	MnO	5.08
P	0.07	S	0.11

**Figure 2:**
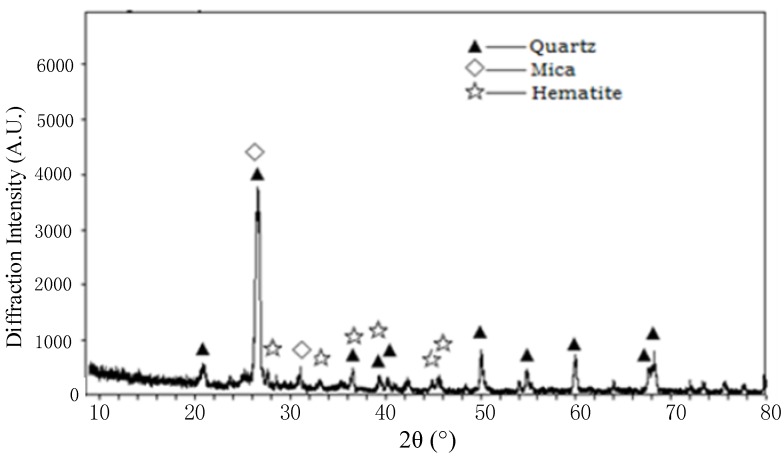
An X-ray diffraction analysis (XRD) of mill tailings.

Based on X-ray diffraction, a mineralogical analysis was conducted (Figure 2). From the peak height of the XRD spectra, the major mineral elements of tailings were quartz, mica, and hematite, whose main chemical components were SiO_2_, Al_2_O_3_, Fe_2_O_3_, and K_2_O, and these analysis results are consistent with those in Table 2.

#### 2.1.2. Binders

Portland cement (PC I) was used as the binder for cemented tailing backfill and was added to the mixture to increase the support potential. The main physical properties are shown in Table 3.

**Table 3:** Main physical properties of Portland cement (PC I).

Item	Value	Item	Value
Fineness (<0.045 mm), %	11.0	28 days flexural strength, MPa	6.6
Initial setting time, min	162	28 days uniaxial compressive strength, MPa	31.5
Final setting time, min	203		

#### 2.1.3. Water

Water from the mine site was added to reach desired consistency, and the chemical properties of water used in cemented tailing backfill were taken into account during the mixture design because this was important for water selection as well as for its direct impact on the mechanical strength development of the backfill body. Table 4 shows the chemical composition of water used to prepare all sample mixtures. As shown in the table, there was a high level of sulphide, indicating a need to consider its effects on the backfill body.

**Table 4:** The chemical composition of water used.

Element	Water (mg/L)	Element	Water (mg/L)
As	0.022	Cl^−^	63.71
Mn	0.52	SO_4_^2−^	742.3
Cu	0.058	PO_4_^2−^	<0.1
Pb	<0.01	HCO_3_^−^	29.4
Zn	0.094	NO_3_^−^	14.14
Cr	0.001		

### 2.2. Testing and Measurement

Because cemented tailing backfill is multi-phase slurry, rheological properties are affected by several factors. In this study, the solid concentration of cemented tailing backfill (PC-1), the tailing/cement ratio (PC-2), the specific weight of slurry (PC-3), and the slump (PC-4) were selected as input factors, and the yield stress (*Y*_1_) and viscosity (*Y*_2_) of cemented tailing backfill were considered as output factors. Tailings in the experiment were obtained from a filling plant, and bulk cement was used as the cementing material. In the experiment, the yield stress and viscosity of cemented tailing backfill were measured using the torque rheometer, and a cylindrical slump container (10 cm in diameter and 10 cm in height) was used to detect the slump of cemented tailing backfill based on solid concentrations ranging from 70% to 80% and tailing/cement ratios being 4, 6, 8, and 10. Table 5 shows the mix proportions and rheological properties of cemented tailing backfill obtained from the experiment.

Rheological properties such as viscosity and yield stress increased with an increase in the solid concentration. However, viscosity and yield stress showed no particular response to the tailing/cement ratio, whereas the slump was affected by the ratio. An increase in the tailing/cement ratio led to a slight increase in the slump. However, this relationship weakened with an increase in the solid concentration.

**Table 5:** Mix proportions and rheological parameters of cemented tailing backfill.

Solid Concentration (%)	Tailing/Cement Ratio	Specific Weight (kg/m^3^)	Slump (m)	Yield Stress (Pa)	Viscosity (Pa·s)
70	4	1.85	0.080	43.85	1.35
70	6	1.84	0.083	48.74	1.51
70	8	1.84	0.085	50.92	1.57
70	10	1.84	0.088	35.62	1.09
72	4	1.89	0.080	73.51	2.27
72	6	1.89	0.080	67.44	2.08
72	8	1.89	0.081	65.50	2.03
72	10	1.89	0.082	66.48	2.05
74	4	1.94	0.060	142.15	4.41
74	6	1.94	0.065	111.05	3.43
74	8	1.94	0.070	80.47	2.48
74	10	1.93	0.073	94.42	2.91
76	4	1.99	0.037	175.96	5.44
76	6	1.99	0.039	177.56	5.46
76	8	1.99	0.040	154.19	4.79
76	10	1.99	0.033	124.95	3.86
78	4	2.04	0.017	300.05	9.38
78	6	2.04	0.019	341.78	10.82
78	8	2.04	0.016	338.25	7.56
78	10	2.04	0.017	322.47	9.24
80	4	2.13	0.012	533.92	12.11
80	6	2.13	0.013	542.38	13.06
80	8	2.13	0.014	521.31	10.97
80	10	2.13	0.012	532.10	12.53

## 3. The PCA Method

### 3.1. The Background of the PCA Method

In complex problems, the PCA method is an effective technique for reducing data dimensions to prevent information repetitions and redundancies to address principal contradictions [34,35,36,37]. The PCA method is a statistical procedure by which an information matrix of possibly correlated original sample data is converted into a set of values for linearly uncorrelated variables known as principal components by using an orthogonal transformation. Without loss of original sample information, the PCA method focuses on the crux of problems not only to reduce information dimensions but also to eliminate any information redundancy and nonlinearity. In this study, a prediction index system for rheological parameters of cemented tailing backfill was developed, and then a data matrix of samples was obtained according to experimental results. Rheological parameters of cemented tailing backfill can be considered a question involving *n* sets of experimental samples and *p* numbers of influencing factors (*p* < *n*) based on the following data matrix:

(1) $$X=\left[\begin{array}{cccc} x_{11} & x_{12} & \cdots & x_{1p}\\ x_{21} & x_{22} & \cdots & x_{2p}\\ \cdots & \cdots & \cdots & \cdots \\ x_{n1} & x_{n2} & \cdots & x_{np} \end{array}\right]$$

where *X* is an *n* × *p* matrix representing *n* sets of data in which each data set consists of *p* variables and *x*_ij_ represents parameters to be tested.

Given dimensional inconsistencies in factors influencing rheological parameters of cemented tailing backfill, and it is necessary to standardize sample data as follows:

(2) $$x_{ij}^{*}=\frac{x_{ij}-\bar{x_{j}}}{\sqrt{var(x_{j})}}$$

where $\bar{x_{j}}$ denotes the mean of the *j*-th variable; $\sqrt{var(x_{j})}$ denotes the standard deviation of the *j*-th variable; *i* = 1, 2, …, *n*; and *j* = 1, 2, …, *p*.

Standardized data were rearranged such that *p* numbers of combined variables were obtained by a linear combination of original variables (*x*_1_, *x*_2_,…, *x*_p_):

(3) $$\{\begin{array}{c} y_{1}=u_{11}x_{1}+u_{12}x_{2}+\cdots +u_{1p}x_{p}\\ y_{2}=u_{21}x_{1}+u_{22}x_{2}+\cdots +u_{2p}x_{p}\\ \cdots \cdots \\ y_{n}=u_{n1}x_{1}+u_{n2}x_{2}+\cdots +u_{np}x_{p} \end{array}$$

where $u_{ij}$ is the loading of the principle component representing the weight of the *j*-th variable projected onto the *i*-th principle component.

In addition, the coefficients $u_{ij}$ had to meet the requirement $u_{k1}^{2}+u_{k2}^{2}+\cdots +u_{kp}^{2}=1$, *k* = 1,2, …, *p*, which were determined by the following principles:

(i) *y*_i_ should be linearly uncorrelated with *y*_j_ (*i* ≠ *j*; *i*, *j* = 1,2, …, *p*).

(ii) *y*_1_ is the first principal component and has the largest variance in a linear combination of *x*_1_, *x*_2_, …, *x_p_*. That is, it accounts for as much variability in data as possible. *y*_2_ is the second principal component and has the largest variance in a linear combination of *x*_1_, *x*_2_, …, *x_p_*, which are linearly uncorrelated with *y*_1_. Similarly, *y_p_* is the *p*-th principal component and has the largest variance in a linear combination of *x*_1_, *x*_2_, …, *x_p_*, which are linearly uncorrelated with *y*_1_, *y*_2_, …, *y_p−_*_1_.

As stated earlier, rearranged integrated variables were defined as the first, second … and *p*-th principal components of original variables such that *y*_1_ had the highest ratio of the total variance and other integrated variables *y*_2_, *y*_3_, …, *y_p_* had gradually decreasing variances. The number of principle components was chosen based on the accumulated contribution ratio of variances such that the larger the accumulated contribution, the less the information loss. However, more calculations were required in both cases. Accordingly, the suitable accumulated contribution ratio was set to approximately 80%, and only several largest principle components were picked for constructing the next model to simplify the structure of the system and grasp the essence of the question.

### 3.2. The Application of the PCA Method for Reducing Rheological Property Data

Although prediction models for rheological properties based on artificial intelligence methods have been introduced [17,18], correlations and overlaps between input data have been ignored, which can have unfavorable effects on the accuracy of prediction models. To overcome this limitation, the PCA method was adopted before constructing prediction models with the BP neural network. New input factors (principal components) were obtained by the orthogonal transformation of original input factors. The number of input factors was reduced effectively almost without any change in original information, and correlations between input factors were eliminated, improving the precision and efficiency of calculations.

Standardized preprocessing [34,35,36,37] was employed using the experimental results for rheological properties of cemented tailing backfill tabulated in Table 6. Then a Pearson correlation analysis was conducted to investigate the interrelationships between four input factors tabulated in Table 7. There were distinct correlations between the specific weight and solid concentration of cemented tailing backfill, as well as between the slump and the solid concentration, indicating the existence of some information overlap between input factors, and therefore the use of principle components was required.

**Table 6:** The standardized processing of index data.

Item	PC-1	PC-2	PC-3	PC-4
1	−1.43303	−1.31339	−1.24818	1.01734
2	−1.43303	−0.43780	−1.35077	1.11851
3	−1.43303	0.43780	−1.35077	1.18596
4	−1.43303	1.31339	−1.35077	1.28713
5	−0.85982	−1.31339	−0.83782	1.01734
6	−0.85982	−0.43780	−0.83782	1.01734
7	−0.85982	0.43780	−0.83782	1.05106
8	-0.85982	1.31339	−0.83782	1.08478
9	−0.28661	−1.31339	−0.32487	0.34286
10	−0.28661	−0.43780	−0.32487	0.51148
11	−0.28661	0.43780	−0.32487	0.68010
12	−0.28661	1.31339	−0.42746	0.78127
13	0.28661	−1.31339	0.18808	−0.43279
14	0.28661	−0.43780	0.18808	−0.36534
15	0.28661	0.43780	0.18808	−0.33162
16	0.28661	1.31339	0.18808	−0.56769
17	0.85982	−1.31339	0.70103	−1.10727
18	0.85982	−0.43780	0.70103	−1.03982
19	0.85982	0.43780	0.70103	−1.14099
20	0.85982	1.31339	0.70103	−1.10727
21	1.43303	−1.31339	1.62434	−1.27589
22	1.43303	−0.43780	1.62434	−1.24216
23	1.43303	0.43780	1.62434	−1.20844
24	1.43303	1.31339	1.62434	−1.27589

**Table 7:** A Pearson correlation analysis of factors influencing rheological properties.

Pearson Correlation Analysis					
Rheological properties		Solid Concentration (%)	Tailing/Cement ratio	Specific Weight (kg/m^3^)	Slump (m)
Solid concentration (%)	Pearson correlation coefficient	1	0.000	0.992 **	−0.969 **
	Significance (two-sided)	0.000	1.000	0.000	0.000
	N	24	24	24	24
Tailing/cement ratio	Pearson correlation coefficient	0.000	1	−0.012	0.041
	Significance (two-sided)	1.000	0.000	**0.957**	**0.849**
	N	24	24	24	24
Specific weight (kg/m^3^)	Pearson correlation coefficient	0.992 **	-0.012	1	-0.955 **
	Significance (two-sided)	0.000	**0.957**	0.000	0.000
	N	24	24	24	24
Slump (m)	Pearson correlation coefficient	−0.969 **	0.041	−0.955 **	1
	Significance (two-sided)	0.000	**0.849**	0.000	0.000
	N	24	24	24	24

Note: ** Distinct correlations between input factors are in bold type.

**Figure 3:**
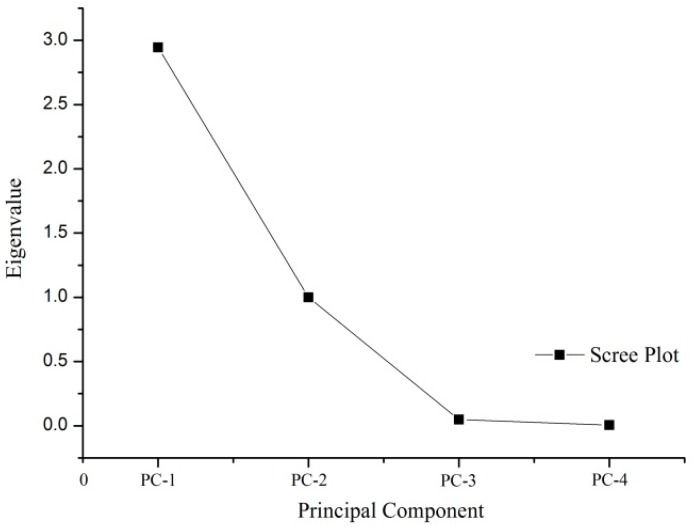
A PCA scree plot.

Analyses were conducted using data in Table 6 based on SPSS (Statistical Package for the Social Sciences) and its PCA function, and principle components and scree plots were obtained. As shown in Figure 3, the X-axis shows principal components sorted by the decreasing fraction of the total variance explained, and the Y-axis shows corresponding eigenvalues. Based on the scree plots, there large differences in eigenvalues between PC-1 and PC-2 and between PC-3 and PC-4, indicating that the first two components of the rearranged information matrix included original information. Therefore, PC-1 and PC-2 were considered the most important indices, and PC-3 and PC-4 were considered non-significant indices. The scree plot was consistent with the cumulative contribution of the first two sets of data in Table 8. Despite some errors in the substitution of two sets of data in the rearranged information matrix for the original four sets of data, test data accounted for 98.634% of information in the original sample, produced reliable calculations, and enhanced computing efficiency because of a reduced number of factors. In addition, the results satisfy the requirement that the ratio of required information accounts for more than 75%–85% of original information in the PCA. Further, the results in Table 8 are consistent with Figure 3.

**Table 8:** Variance and cumulative contributions of PC-1, PC-2, PC-3, and PC-4.

Components	Initial Eigenvalue			Accumulated Contributions		
	Total	Variance (%)	Accumulation (%)	Total	Variance (%)	Accumulation (%)
PC-1	2.945	73.622	73.622	2.965	73.622	73.622
PC-2	1.000	25.012	98.634	1.000	25.012	98.634
PC-3	0.048	1.210	99.844			
PC-4	0.006	0.156	100.000			

A coefficient matrix of principle components was calculated and tabulated based on SPSS in Table 9 for relationships between two principle components and original data:

(4) $$Z_{1}=0.338\times \text{PC}1-0.009\times \text{PC}2+0.337\times \text{PC}3-0.334\times \text{PC}4$$

(5) $$Z_{2}=0.027\times \text{PC}1-0.999\times \text{PC}2+0.015\times \text{PC}3-0.016\times \text{PC}4$$

Principal components (PCs) were determined using the standardized data in Table 6 according to the aforementioned formula. The prediction model was constructed using the results tabulated in Table 10.

**Table 9:** The weight factor matrix of principle components.

Coefficient	Principle Component	
	Z_1_	Z_2_
PC1	0.338	0.027
PC2	0.009	0.999
PC3	0.337	0.015
PC4	0.334	0.016

**Table 10:** Experimental data with the PCA method.

No.	Z_1_	Z_2_	Yield Stress (Pa)	Viscosity (Pa·s)
1	−1.23297	1.27439	43.85	1.35
2	−1.30922	0.399356	48.74	1.51
3	−1.33962	−0.47488	50.92	1.57
4	−1.3813	−1.34893	35.62	1.09
5	−0.90094	1.290709	73.51	2.27
6	−0.90882	0.416121	67.44	2.08
7	−0.92796	−0.4583	65.50	2.03
8	−0.9471	−1.33271	66.48	2.05
9	−0.30905	1.30441	142.15	4.41
10	−0.37325	0.430723	111.05	3.43
11	−0.43745	−0.44297	80.47	2.48
12	−0.51369	−1.31801	94.42	2.91
13	0.31663	1.31757	175.96	5.44
14	0.286221	0.443342	177.56	5.46
15	0.267078	−0.43108	154.19	4.79
16	0.338045	−1.30693	124.95	3.86
17	0.908515	1.331271	300.05	9.38
18	0.878106	0.457043	341.78	10.82
19	0.904017	−0.4181	338.25	7.56
20	0.884874	−1.2925	322.47	9.24
21	1.469734	1.351618	533.92	12.11
22	1.450588	0.47721	542.38	13.06
23	1.431445	−0.39721	521.31	10.97
24	1.446093	−1.27216	532.10	12.53

## 4. The Prediction of Rheological Properties of Mining Cemented Tailing Backfill

### 4.1. The Prediction Model Combining the PCA Method with the BP Neural Network

**Figure 4:**
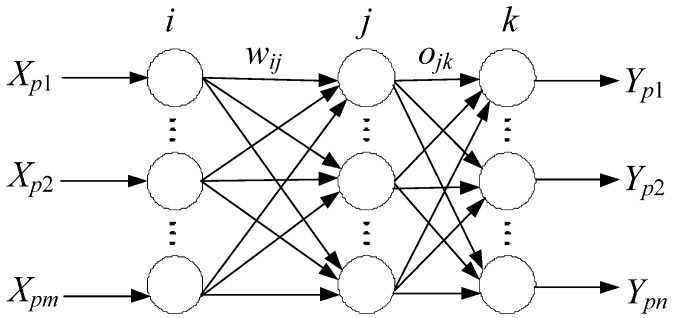
The topological structure of a three-layer BP neural network.

The BP learning algorithm can be divided into two phases: The transmission of operating signals (forward propagation) and the back-propagation of errors [38,39,40]. During the forward propagation of operating signals, the input signal propagates from the input layer through a hidden layer to the output layer, and the status of each layer of neurons influences only the next layer of neurons. If the output cannot be achieved in the output layer, then it should be switched to the back-propagation process of error signals. During the back-propagation of error signals, the error signal propagates from the output end to the input layer in a layer-by-layer manner, and the weight of the network is regulated by error feedback. With the adoption of the gradient descent method in the weighted vector space, the continuous modification of weight and offset values is applied during dynamically interactive searches, making the real output of the network closer to the expected one to achieve information extraction and memory, as shown in Figure 4.

Given a set of *N* samples (*X*_K_, *Y*_K_) (*k* = 1,2, …, *N*) corresponding to the *j*-th unit in the *l*-th layer, the output of the *j*-th node with the input of *k* samples can be described as

(6) $$net_{jk}^{l}=\sum_{j}W_{ij}^{l}O_{jk}^{l-1}$$

where $W_{ij}^{l}$ represents the weight and $O_{jk}^{l-1}$ represents the output of the *j*-th node in the (*l*−1)-th layer when the *k*-th sample is imputed.

The output of the *l*-th node can be expressed as $O_{jk}^{l}=f(net_{jk}^{l})$ such that the sigmoid function is adopted as the activation function *f*:

(7) $$f\left(x\right)=\frac{1}{1+e^{-x}}$$

The error function can be expressed as

(8) $$E_{K}=\frac{1}{2}{\sum_{i}\left(Y_{jk}-\bar{Y_{jk}}\right)}^{2}$$

where $\bar{Y_{jk}}$ is the actual output of the *j*-th unit such that the total error can be calculated by

(9) $$E=\frac{1}{2N}\overset{N}{\underset{k=1}{\sum }}E_{k}$$

If $\delta_{jk}^{l}=\frac{\partial E_{k}}{\partial net_{jk}^{l}}$, then the steps of the algorithm can be described as follows:

Step 1: The initial weight is assigned randomly.

Step 2: The following processes are repeated until E<ε, where ε is predetermined precision:

(i) Calculations for *k* = 1 to *N*:

The forward propagation of the input signal: $O_{jk}^{l}$, $net_{jk}^{l}$, and $Y_{jk}$ of each unit with *k* = 2, …, *N*;

The back-propagation of the error signal: $\delta_{jk}^{l}$ of each unit in the hidden layer.

(ii) The weight correction:

(10) $$W_{ij}=W_{ij}-\mu \frac{\partial E}{\partial W_{ij}},\;0<\mu <1$$

(11) $$\frac{\partial E}{\partial W_{ij}}=\sum_{k=1}^{N}\frac{\partial E_{k}}{\partial W_{ij}}$$

**Figure 5:**
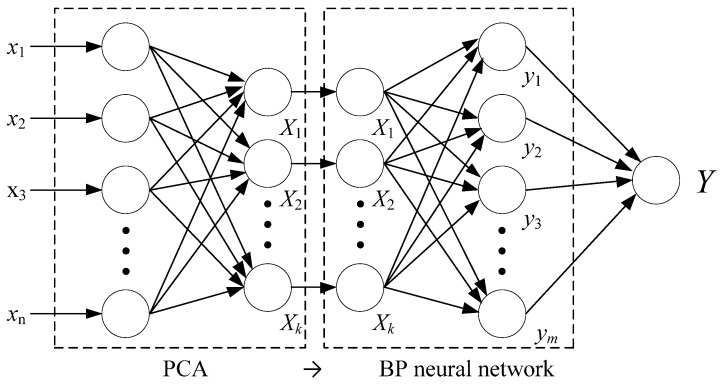
The prediction model based on the BP neural network combined with the PCA method.

First, a set of correlated variables is transformed into a set of linearly uncorrelated variables called principle components obtained from the PCA such that obtained variables are adopted as input variables in the BP neural network, as shown in Figure 5.

### 4.2. The Design and Training of the Prediction Model

The network topology and calculation procedures introduced in the previous section were adopted. X1 and X2 were employed as input factors, and the yield stress and viscosity of cemented tailing backfill were employed as output factors. Experimental data from the PCA tabulated in Table 10 were divided into two groups: A training data set (1–20) and a prediction sample set (21–24). For the construction of the prediction model, the learning rate and the momentum coefficient were set to 0.972 and 0.8, respectively.

A three-layer network was adopted in the optimization calculation such that the node numbers were 7, 15, and 2. MATLAB was used to obtain the prediction results tabulated in Table 11 and Table 12. The prediction error for the BP neural network model combined with the PCA method was less than 5%. In sum, the BP neural network prediction model combined with the PCA method clearly enhanced predictive accuracy in comparison to conventional ANNs with no PCA. The PCA method could identify the correlations between input variables and reduced the number of input variables, enabling the construction of an accurate prediction model.

**Table 11:** Comparisons of predicted yield stress between the PCA model and the non-PCA model.

No.	Expected Value of Yield Stress (Pa)	PCA Model		Non-PCA Mode	
		Predicted Value with the BP Neural Network	Relative Error (%)	Predicted Value with the BP Neural Network	Relative Error (%)
21	533.92	514.86	3.57	475.94	10.86
22	542.38	537.93	0.82	503.55	7.16
23	521.31	513.28	1.54	476.95	8.51
24	532.10	506.93	4.73	504.38	5.21

**Table 12:** Comparisons of predicted viscosity between the PCA model and the non-PCA model.

No.	Expected Value of Viscosity (Pa·s)	PCA Model		Non-PCA Model	
		Predicted Value with BP-ANN	Relative Error (%)	Predicted Value with rBP-ANN	Relative Error (%)
21	12.11	11.61	4.17	11.40	5.84
22	13.06	12.89	1.33	12.12	7.16
23	10.97	10.69	2.54	10.04	8.51
24	12.53	12.42	0.89	17.77	6.09

## 5. Conclusions

It is important to characterize the interrelationships between paste components and rheological properties to optimize the workability of cemented tailing backfill. In this regard, experiments were conducted to examine rheological properties of cemented tailing backfill by considering the solid concentration and the tailing/cement ratio. Intercorrelations between the content of components and rheological properties were comprehensively investigated. An increase in the solid concentration increased rheological properties of cemented tailing backfill. Rheological properties and the specific weight increased with an increase in the solid concentration. However, rheological properties showed no particular response to the tailing/cement ratio, whereas the slump was affected by the ratio.

Based on the experimental results, a prediction model was constructed. More specifically, the BP neural network was combined with the PCA method to predict rheological properties of cemented tailing backfill. The PCA method could identify intercorrelations between input variables and reduced the number of input variables, making it possible to construct an accurate prediction model with no changes in major information in sample data. That is, the proposed approach accurately and quickly predicted rheological properties through a simple network structure. The BP neural network prediction model combined with the PCA method clearly enhanced predictive accuracy in comparison to conventional ANNs without the PCA method. Validation results show that the error between estimated and output values obtained using the proposed prediction model was within 5%, reflecting a remarkable improvement over that for conventional ANN prediction models without the PCA method.
